# Patient Experience of Virtual Hospital Care Provided by a Multidisciplinary Team: Protocol for a Mixed Methods Study

**DOI:** 10.2196/72729

**Published:** 2025-08-15

**Authors:** Tim M Jackson, Kanesha Ward, Saranjit Singh, Rezwanul Rana, Winnifred Li, Chenyao Yu, Jason Levy, Michelle Chen, Mui Chung, Shelley Somi, Kim Offner, Enrico Coiera, Annie AY Lau

**Affiliations:** 1 Australian Institute of Health Innovation Sydney Australia; 2 Northern Sydney Local Health District Sydney Australia

**Keywords:** co-design, health economics, multidisciplinary teams, recommendations, remote monitoring, virtual care, virtual hospitals

## Abstract

**Background:**

Virtual hospitals are defined as the delivery of hospital-level health care via digital services such as videoconferencing technologies, digital platforms, and remote monitoring. The purpose is to leverage virtual technologies to deliver accessible patient-centered care, improve the overall health of communities, and implement improvements driven by real-time access to patient data. Patients and their caregivers have increasingly favored these alternative and complementary service delivery models alongside traditional in-person care. However, this is still a complex issue, and the current literature points to a variety of challenges that need to be overcome to provide optimal models of care.

**Objective:**

This study aims to identify unique challenges to virtual hospitals providing multidisciplinary care for patients at home, and co-design recommendations to improve the patient experience.

**Methods:**

This research is a mixed methods exploratory case study of a virtual hospital in Sydney, New South Wales, Australia. The methods include (1) document analysis: this will be used to identify the current formal processes that govern the virtual hospital; (2) secondary analysis of data: a detailed investigation of existing data collected by the virtual hospital, for example, patient-reported experience measures, patient-reported outcome measures, and other available data; (3) observations of current practices; (4) semistructured interviews; (5) co-designed focus groups; (6) economic analysis; (7) comparative case study; and finally, (8) a triangulation analysis. To synthesize these findings within a unified analytical framework, all data will be subjected to open or axial coding following the Strauss framework, allowing the convergence of themes across codes and methods.

**Results:**

As of April 2025, observations and initial documentation analysis have begun. Multidisciplinary team meetings and clinician shadowing have commenced, along with analysis of Standards of Practice documents.

**Conclusions:**

This protocol outlines a mixed methods case study on a new virtual hospital located in Sydney, New South Wales, Australia. We anticipate our results to provide a comprehensive understanding of patient experience through a range of quantitative and qualitative research activities. We will achieve this by identifying challenges for patients, carers, and health care workers, and documenting informal solutions discovered through our research activities.

**International Registered Report Identifier (IRRID):**

DERR1-10.2196/72729

## Introduction

### Overview

Virtual hospitals are complex, emerging health care initiatives that remotely provide health care to patients. They achieve this via digital services such as videoconferencing technologies, digital platforms, and remote monitoring [1,2]. The purpose is to leverage digital technologies to deliver hospital-level care to patients at home [3]. Increasingly, patients and their caregivers have favored these new virtual care alternatives and complementary service delivery models alongside traditional in-person care [4]. However, virtual hospitals are still in their infancy, and the current literature points to a variety of challenges that need to be overcome to provide optimal models of care [5-13]. The goal of this research is to identify the challenges of an emerging Australian virtual hospital and develop novel recommendations to improve the patient experience.

Of the variety of challenges patients of virtual hospitals face, one example is the difficulty in building clinician-patient rapport through virtual communication [14]. This was highlighted in a study of a virtual rural service in New South Wales (NSW), where there was a desire for continuity of care and how it was difficult to accommodate patients’ preference for “a doctor who knows me” [15]. Another study, a review of telehealth in emergency medicine in Australasia, investigated the difficulties of ensuring the safety of virtual hospital patients [16]. Specifically, when and how to escalate for help, or ways to conduct safe and accurate physical examinations [8]. This challenge is concerning considering the technological barriers patients might encounter when accessing virtual care [5-7]. However, virtual care driven by mobile conferencing technologies can be highly supportive to assist low-staffed emergency departments (EDs). These “virtual care carts” provide an effective communication tool for health care workers, but their use raises questions about appropriateness and lack of privacy when using such technologies in crowded rural emergency rooms [15]. In addition, there has been an increase in additional responsibilities shifted to patients when using virtual care [9-11] (such as setting up remote monitoring technologies at home), calling for further research into the impact of unintended burdens on carers and family support systems [11,12].

The literature also highlights challenges from the perspective of virtual hospital health care workers. One study on a virtual rehabilitation service found that the medical complexity of patients, such as patients with heart failure and cognitive impairments, can impact a health care worker’s confidence in virtual care delivery [17]. This can be exacerbated with virtual care’s disruption to the clinical culture of a health care team. For example, an interview study of health care workers from 3 NSW virtual hospitals found that the shift to virtual care required a time-consuming and extensive adoption process [18]. This is in addition to the shift in responsibilities for health care workers, requiring further staff training to increase the efficiency, reach, and utility of available virtual hospitals [9-11,17]. Finally, there is a lack of studies and tools available to health care workers to help assess whether patients (and their carers) are appropriately referred to and supported during virtual care [6,11,13].

Virtual hospital solutions, such as the Virtual Care Services (VCS) in the Northern Sydney Local Health District (NSLHD), have made great strides in providing remote health care. In particular, NSLHD VCS uses digital technologies to empower a multidisciplinary approach (eg, collaborative care from nurses, doctors, allied health, social workers, etc) to provide hospital-level care to patients at home. This protocol analyzes and improves the benefits of NSLHD VCS by identifying challenges patients, carers, and health care workers encounter and co-designing practical solutions to improve the patient experience. These perspectives will be analyzed according to the process, clinical, and economic stances, and compared between (1) different timepoints (eg, before and after introduction of VCS), (2) virtual and in-person services, (3) Work-As-Imagined (WAI) and Work-As-Done (WAD), and (4) different stakeholder viewpoints. In addition, the research will conduct a comprehensive economic analysis to assess the economic impact of virtual care on patients, carers, health care workers, and NSW Health. This includes identifying financial implications and the economic impact of implementing and operating VCS from NSLHD’s perspective. This study also answers the call for a more rigorous analysis of virtual care solutions using a multimethod approach [19], aiming to co-design practical recommendations to enhance patient safety, improve quality of care, and optimize health care service provision [20].

### Study Setting

This study examines the launch of NSLHD’s VCS. The goal of the service is to support virtual health care in the NSLHD, which was created in response to the growing demand for health care services in NSW [21]. Physically located in Royal North Shore Hospital, this new service is part of the NSW Health Digital strategy and is a key initiative to facilitate and support the delivery of virtual care to patients and the community across the NSLHD [22]. The services include several health condition–specific models of care, including but not limited to the following:

1. AvoidED: This is an integrated formalized pathway in which NSW Ambulance clinicians can safely access a centralized point of contact for clinical discussion and referral of clinically appropriate patients to NSLHD VCS to reduce preventable presentations to NSLHD EDs.
2. Hyperemesis gravidarum model of care: This is an assessment and care model to provide referral of care between hospital- and community-based services for women impacted by hyperemesis gravidarum.
3. Frailty model of care: An evidence-based 2-week program for older patients who have been assessed as “Frail” at hospital discharge provides follow-up care to reduce the possibility of ED representation within 4 weeks of hospital discharge. This program provides patients with (1) a follow-up on the hospital discharge plan, (2) safety netting and escalation of care as appropriate, (3) virtual physiotherapy services at home, and (4) referral and linkage to community-based services for long-term frailty management.

**Figure 1:**
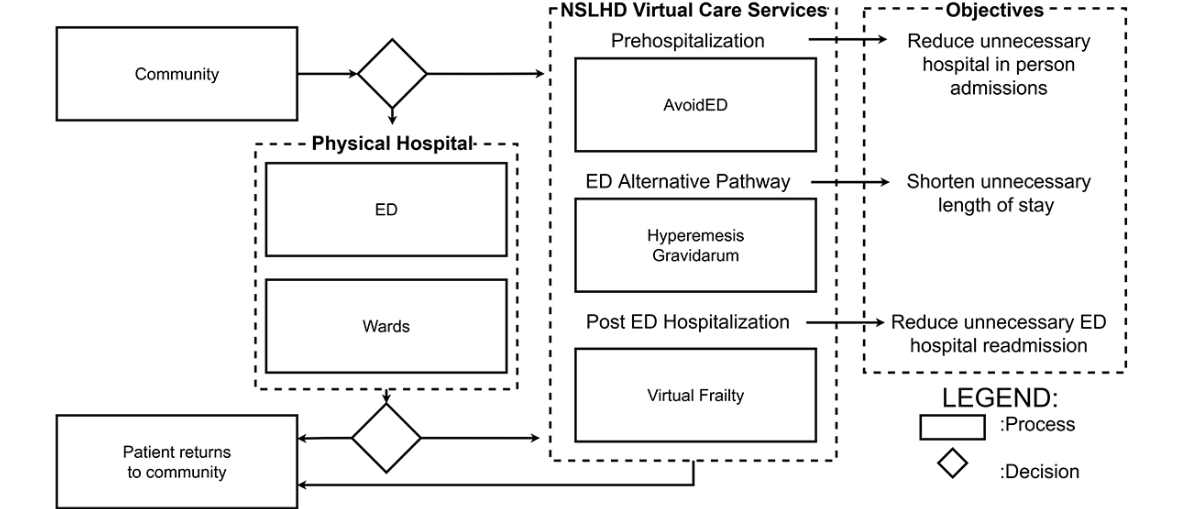
Virtual hospital summary diagram. ED: emergency department; NSLHD: Northern Sydney Local Health District.

The NSLHD VCS was created to support existing health care services and facilitate access to hospital-level care in a patient’s home. Figure 1 depicts a summary overview of the process of how a patient might interact with NSLHD VCS. The alternate pathway choices are by referral from clinicians.

### Aim

The primary aim of this study is to evaluate models of care within the NSLHD VCS from the patient’s perspective. We will investigate challenges encountered by patients and carers via exploring their experience, identifying existing informal solutions, and collaboratively co-designing recommendations to improve the service. Our secondary aim is to identify these services’ clinical outcomes and economic impact. By understanding the financial implications of this service, NSW Health can be better equipped in its decision-making process to provide the best possible care for the patient.

## Methods

### Research Activities

Considering the novel approach of NSLHD VCS [23,24] and its complex structure [25], this study will use an exploratory mixed methods case study. The purpose of using this approach is to use established mixed methodologies to construct a quality [26], rigorous [27], and credible study [28].

The study was built on a series of stages: (1) document analysis, (2) secondary analysis of data, (3) observations, (4) semistructured interviews, (5) co-designed focus groups, (6) economic analysis, (7) comparative case study, and (8) a triangulation analysis. The expected outcome of this study is a comprehensive understanding of how NSLHD VCS operates [29] and the production of a practical list of recommendations for its improvement [30].

This research protocol and methodology have been presented to a 10-member consumer panel, where it was received positively. Their feedback has been incorporated in shaping our research questions, research activities, and data analysis strategies.

### Stage 1: Document Analysis

This initial stage of research aims to identify any documentation that will allow the research team to better understand how these models of care function at the NSLHD VCS (eg, WAI). This includes a review of documents such as (but not limited to): referral and assessment forms, care escalation protocols, internal documents (eg, Standards of Practice [SOP], resources involved, system use, and time spent on various activities), working group documents, official escalation processes, and other publicly accessible documents. The analysis will be guided by Altheide and Schneider’s 12-step and 5-stage process (eg, document sampling, protocol development and data collection, data coding and organization, data analysis, and reporting) [31]. Depending on the focus of our investigation, we may consider conducting content or thematic analysis, where an inductive approach will be applied to identify WAI aspects (eg, patient safety and care escalation) contained in the documents.

These data will also be used to inform the study design of prospective data collection approaches (observations, interviews, and focus groups), and as a secondary method in future triangulation of data analysis with the other research activities to contextualize and understand WAI.

### Stage 2: Secondary Analysis of Data

The secondary analysis refers to data previously collected by the NSLHD VCS as part of routine procedures and processes. This can include (but is not limited to): (1) patient-reported experience measures; (2) patient-reported outcome measures; (3) administrative data, such as the number of referrals, admission rates, number of separations, and discharge summary reports; (4) process-specific data, such as a model of care workflows and admission summaries; (5) electronic medical record data, such as admission rates, length of stay, demographics, condition type, severity level, and tool scores; and (6) Incident reporting, such as the number of escalations and adverse event reports.

Descriptive analysis will be conducted on this routinely collected deidentified data. These data will also be used to inform the study design of prospective data collection approaches (eg, observations, interviews, and focus groups).

### Stage 3: Observations

To assist in the development of a detailed understanding of the function of NSLHD VCS (ie, WAD), the research team will conduct a series of observations of the processes involved in models of care and virtual consultations [32]. Through direct observation, we hope to identify elements of care that interviewees might have omitted due to familiarity with the context [33]. Observations shall also assist in understanding the complex relationships between health care staff and recipients of services (eg, patients and their carers), along with the detailed scenarios that occur within clinical environments [34]. This is also referred to as the sociomaterial perspective*,* which is used to help understand interactions between systems, materials, places, humans, and nonhuman actors that do not easily respond to methods such as surveys and interviews [35]. Observation methodology is chosen considering that real-world behavior is difficult to capture in a research setting [36], where observational data not only minimize self-reporting bias [37] but also provide greater insight into the patient experience at different stages of their virtual hospitalization journey [38].

Observational data collection tools are considered but are not limited to the following:

- Descriptive fieldnotes: the observational data created will include (but are not limited to) descriptive fieldnotes from multiple observers [32] and analytical notes to capture the context of consultations [36].
- Semistructured templates: we have adapted a semistructured template from the work of Fix et al [36]. Here we use a mix of structured and open-ended inputs to capture detailed and consistent observational data. Observations related to constructs informed earlier by Stage 1 WAI document analysis will be recorded. Other observations that have not been previously identified will be recorded in the open-ended free-text section of the semistructured form. For example, to structure the observations of how health care workers interact with the technologies of the virtual hospital, the semistructured template will be based on the IS success model [39]. Here, the constructs will include system quality, information quality, system use, user satisfaction, individual impact, and organizational impact (Multimedia Appendix 1).

The objective of this research activity is to observe multidisciplinary team (MDT) meetings (ie, group meetings), clinician-patient consultations, clinician-IT human-computer interactions, and clinician-clinician interactions. A total of 2 graduate students have been trained to use the semistructured templates and have weekly debriefs to discuss the implications of the observations. These observations are also overseen by 2 senior academics to help interrater reliability.

Observers will have a passive role, meaning they shall not have any clinical intervention or impact on the clinical workflow of the VCS. The goal is to reduce bias through unobtrusive strategies to limit the impact of the researcher on the research environment [40] and provide context-rich information that is difficult to discern through other research activities [32].

The data collected in this stage will be analyzed using conventional data analysis approaches [41], such as narrative, content, or thematic analysis [32,40,42]. Each of these analysis techniques will be triangulated with data from the experience of the observer. This will aid in the interpretation of the data to yield a richer, more representative analysis [43].

### Stage 4: Semistructured Interviews

Semistructured interviews will be conducted with patients, carers, and health care workers (ie, clinical and nonclinical staff members of NSLHD VCS). To reach saturation of knowledge [44,45] and the heterogeneity of the sample size [46], we aim to recruit 20 participants from each stakeholder group. This is based on recommendations from the literature that each participant group of a case study should number from 5 to 25 participants [47] or at least 6 [48]. Once the interviews are completed, 2 investigators will review and analyze each of the transcripts created to begin to identify emergent themes [42,49]. Interview transcripts will then be analyzed through thematic and content analysis [42].

### Stage 5: Co-Designed Focus Groups

Focus groups will be organized with patients, carers, and health care workers (ie, clinical and nonclinical staff members of NSLHD VCS) using an experience-based co-design approach [50]. The goal here is to unify the data collected and analyzed in the previous stages of this research. Here, different stakeholder groups can be presented with significant interactions identified within NSLHD VCS [51] and collaboratively create recommendations for the improvement of health care services [52]. The health care co-design framework of Boyd et al [50] will be used to guide the focus groups and the evaluation process.

### Stage 6: Economic Analysis

A comprehensive analysis will be conducted on the economic effects of VCS from different stakeholder perspectives. Past economic research has assessed VCS within existing health care systems using well-established health economic methods, including cost-effectiveness, cost-consequence, and cost-utility analyses [53,54]. Much of the existing health economic research relies on quantitative data [53], mainly direct costs to the health system [54], but overlooks patient and carer cost impacts (especially from a qualitative perspective) [55]. Depending on study feasibility, time constraints, access to resources, as well as data availability, we aim to collect perspectives from 3 different types of stakeholders, namely:

- The health system perspective: this perspective considers costs incurred by the health care system, including government health care funding, hospitals, and insurers. It includes direct medical costs such as hospital stays, medical procedures, medications, and health care professional fees.
- The patient and carer perspective: this is the broadest approach, capturing both direct and indirect costs. It includes patient and caregiver expenses, such as out-of-pocket costs, travel, accommodation costs, lost wages due to illness, quality-of-life changes, and informal caregiving.
- The health care worker perspective: this focuses on costs experienced directly by health care workers when working in a virtual hospital environment. It includes out-of-pocket expenses, technology investments (eg, computer costs, internet bills, and electricity costs), and quality-of-life changes.

The goal of the economic analysis would allow the exploration of direct costs, indirect costs, and outcomes without aggregating them into a single measure, providing a detailed view of the effects of VCS models of care. Building on insights from both patients and providers, we will explore different dimensions that outline the diverse value of virtual health care economic assessment. Here, we will determine the impact of VCS using metrics of use, costs, productivity, and health outcomes (where data are available) [56].

### Stage 7: Comparative Case Study

This stage aims to evaluate the impact of the NSLHD VCS through examination of clinical, process, and economic outcomes. This stage will help inform future VCS implementation using qualitative and quantitative methods. The comparison will include (but is not limited to):

- Different timepoints: evaluate changes in patient experience, clinical outcomes, and economic impacts before and after the introduction of VCS.
- Virtual and in-person service: understand the variations in patient journeys, clinical effectiveness, and economic implications (such as direct and indirect costs) by comparing the 2 modes of care delivery.
- WAI and WAD: examine how planned processes in NSLHD VCS align with actual implementation and practice to understand operational challenges and the implementation of virtual models of care.
- Different stakeholder viewpoints: understand the perspectives of patients, carers, and health care workers regarding VCS to identify areas for improvement and the value of VCS.

### Stage 8: Triangulation Analysis

**Figure 2:**
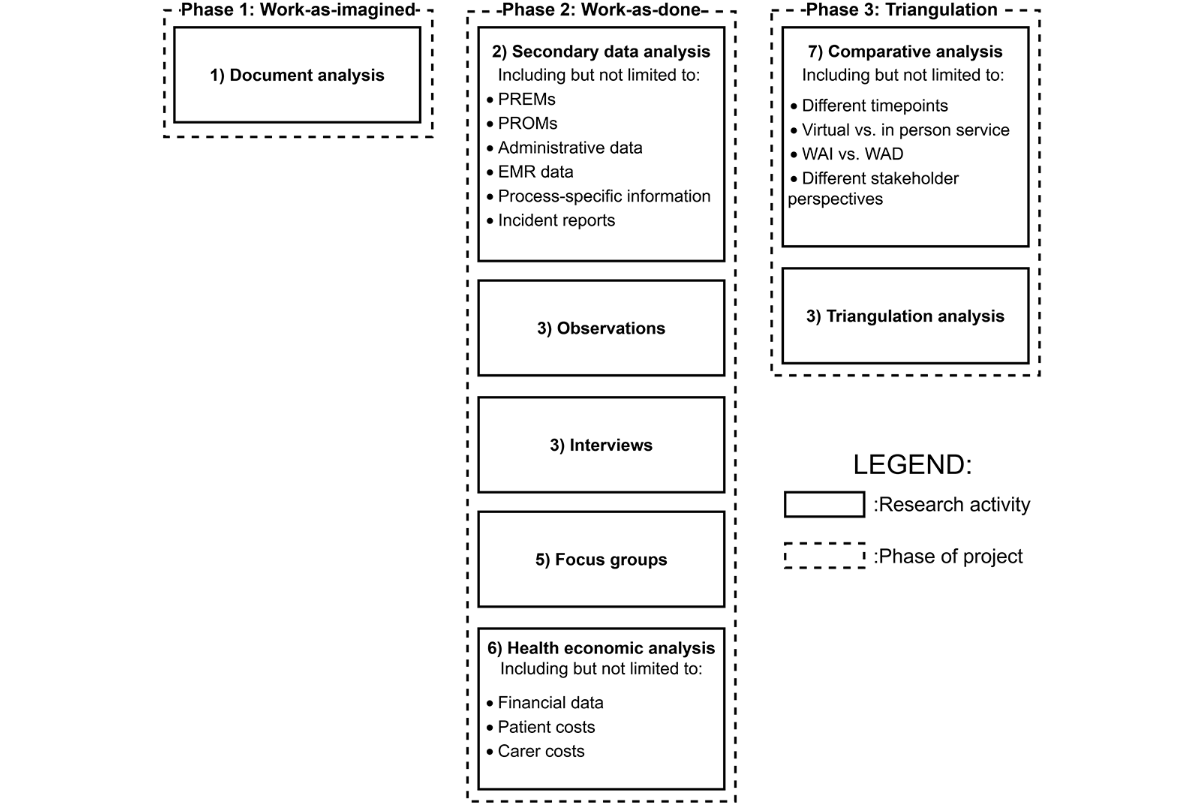
Phases of the research project. EMR: electronic medical record; PREM: patient-reported experience measure; PROM: patient-reported outcome measure; WAD: Work-as-Done; WAI: Work-As-Imagined.

The final stage is a triangulation analysis of each of the previous stages’ data (ie, method triangulation) [57]. The purpose is to synthesize all research activities to develop a comprehensive understanding of the patient experience [26] and increase the level of scientific rigor [58]. Here, we can build a “more detailed and balanced picture of the situation…” where “The contradictions which are often hidden in situations become visible, enabling a more profound interpretation.” (p. 117) [59]. The triangulation analysis is represented in a series of phases (Figure 2):

- Phase 1: WAI. This is the exploration of internal documents to understand the formal structure of VCS and the intentions of use.
- Phase 2: WAD. This is the realistic process of the VCS and how it currently operates. This phase will combine observational, interview, and focus group data.
- Phase 3: Triangulation. This is the complete exploration and comparison of all the research activities. The triangulation method begins with an analysis of each of the different categories of data separately. As Flick [60] outlines, the commonalities and differences are identified (eg, between the WAI and WAD), as well as recognizing broader patterns and the validation of results. To synthesize these findings within a unified analytical framework, all data will be subjected to open or axial coding following the study by Strauss et al [61], allowing for the convergence of themes across codes and methods. This consistent approach to coding allows for the integration of themes and insights across datasets, for a more robust and holistic presentation of results.

Explorative and iterative analysis methods will be used to create comparisons between interactions, perspectives, and timepoints in patients’ experiences with VCS. This shall build a comprehensive picture of patient experience and identify where additional support may be provided to patients, health care workers, or their carers.

### Participant Recruitment

Primarily, we are seeking to recruit patients, carers, and health care workers at NSLHD VCS. Health care workers (clinicians and nonclinicians) used at NSLHD VCS, as well as patients and carers who have used these virtual hospital services, will be approached to participate in the study. Participants will be included in this study if they meet the following inclusion criteria: (1) they have interacted with the NSLHD VCS; this can include employment, receiving health care, or assisting another receiving health care at the NSLHD VCS; (2) they are older than 18 years; and (3) they have consented to be part of the study.

Health care workers will be recruited through email and education sessions. The goal of the education sessions is to explain the research aims and methodologies, how patient and carer recruitment works, and how health care workers can help and participate.

Patients and carers will be recruited prospectively as well as retrospectively. NSLHD will advertise our study to currently admitted and discharged patients and carers. We will apply inclusion and exclusion criteria for any individuals who contact us or who have provided permission for researchers to contact them. Patients and carers completing a 40-minute interview or a 60-minute focus group will be compensated for their time (a US $26.01 gift voucher for the interview and a US $39.01 gift voucher for the focus group). Any patient or carer participant who is interviewed and participates in a focus group will be given both compensation amounts (ie, a single US $65.02 gift voucher).

Researchers will send emails and SMS reminders to all potential participants (up to 3 times over 2-4 weeks). If there is no response after following up, researchers will assume the individual does not wish to participate and cease follow-up contact.

### Prospective Patient and Carer Recruitment

Prospective patients are those who are currently admitted to the NSLHD VCS. They can be recruited via these two approaches: (1) NSLHD health care workers can inform patients and their carers about the study during their routine consultation, seeking permission for researchers to contact them about the research; or (2) A NSLHD VCS team can also send an email and SMS to currently admitted patients.

The email and SMS will contain links to the study Participant Information Sheet (PIS), Participant eConsent form, and research team contact details. Patients and carers can then make an informed decision by reading the PIS and contacting the research team. They can also directly enrol in the study via an eConsent form.

### Retrospective Patient and Carer Recruitment

**Figure 3:**
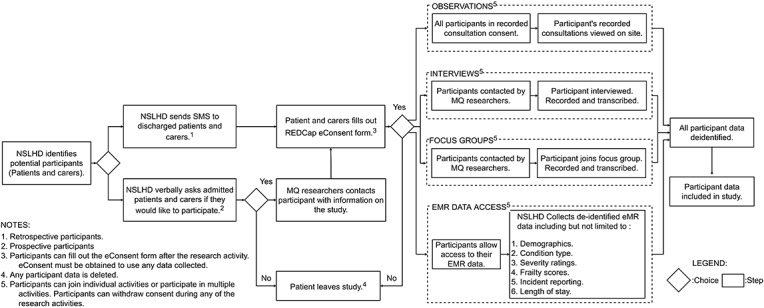
Patient and carer recruitment and consent process. EMR: electronic medical record; MQ: Macquarie University; NSLHD: Northern Sydney Local Health District; REDCap: Research Electronic Data Capture.

Retrospective patients are those who have been discharged from NSLHD VCS before the start of the study. The NSLHD VCS team will send an email and SMS to retrospective patients, containing a link to the PIS, Participant eConsent form, and the research team contact details. Patients can then make an informed decision by reading the PIS and contacting the Macquarie University research team. They can also directly enroll in the study via an eConsent form via email and SMS. Refer to Figure 3 for a summary of the patient and carer recruitment and consent process.

### Ethical Considerations

Ethical approval was granted by the Macquarie University Human Research Ethics Committee for Medical Sciences (reference 520241860460310) and the Northern Sydney Local Health District Ethics Review Committee (2024/ETH01430).

## Results

As of February 2025, all required ethics applications from NSLHD have been approved and site-specific permission has been granted. Observations and initial documentation analysis have begun. Observations of MDT meetings, clinician shadowing, and analysis of the SOP documents have commenced. We have also begun education sessions for health care workers, commenced recruiting health care workers, and advertised the study to a range of patients and their carers at NSLHD VCS.

## Discussion

### Principal Findings

This protocol outlines an exploratory mixed methods case study on a new virtual hospital located in Sydney, NSW, Australia. We anticipate our results will provide a comprehensive understanding of patient experience and virtual hospitalization, specifically for those receiving hospital-level care at home, facilitated by a multidisciplinary health care team and supported by digital technologies. Through a range of quantitative and qualitative research activities, we will identify challenges encountered by patients, carers, and health care workers, and co-design a list of practical and evidence-based recommendations to improve the patient experience.

### Strengths and Limitations

The strengths of this study stem from the diverse research group and extensive research activities used to study the VCS. However, this study will be limited by the extent of access to patient data. The decision not to access the entirety of patient medical records was first based on the risk to the patient’s confidentiality, and second, that deidentified records currently requested will provide ample empirical data of the patient’s experience.

### Comparison With Prior Work

The significant demand for VCS like NSLHD VCS has seen a rise in mixed methods protocols in Australia and abroad. For example, new protocols have been published on the evaluation of a virtual triage service in Victoria [62] and another on the analysis of an acute respiratory model of care in NSW [3]. Internationally, a recent protocol was published for a pilot breast cancer support application in Copenhagen [63] and another for the launch of a new Diabetes-focused hospital information system in India [64].

However, to the best of our knowledge, few published works and protocols endeavor to explore a multidisciplinary virtual service like NSLHD VCS. The literature referred to above generally has a limited focus and lacks the comprehensive research activities of this protocol. In addition, few include an economic evaluation [3] or a direct comparison of patient experience and the financial costs of virtual services.

### Conclusions

This protocol outlines a new collaborative research project to study the NSLHD’s VCS. This virtual hospital is an MDT that leverages digital technologies to provide hospital-level care for patients at home. Through a combination of methodologies, our study will provide a comprehensive understanding of the patient experience of virtual hospitals, identify challenges, and co-design a list of practical and evidence-based recommendations to improve the service.

## Appendix

Multimedia Appendix 1 Observation template.

## Abbreviations

ED: emergency department
MDT: multidisciplinary team
NSLHD: Northern Sydney Local Health District
NSW: New South Wales
PIS: Participant Information Sheet
SOP: Standards of Practice
VCS: Virtual Care Services
WAD: Work-As-Done
WAI: Work-As-Imagined
