# Supplementary material for: Patient Experience of Virtual Hospital Care Provided by a Multidisciplinary Team: Protocol for a Mixed Methods Study
Source: JMIR Res Protoc. 2025 Aug 15;14:e72729. doi: 10.2196/72729 (PMC12397751; doi:10.2196/72729)
Supplement: Multimedia Appendix 1 [file resprot_v14i1e72729_app1.docx]

| Semistructure Observation Template | | | | | | |
| --- | --- | --- | --- | --- | --- | --- |
| Setting | *Eg, routine consultation* | Time start and stop: | | *Eg, 1300-1400* | Date |  |
| Recorder | *Eg, investigator 1* | ID | | *Eg, observation1.* |  |  |
| Participant #1 Type: *Eg, health care worker (Nurse).*  Participant #2 Type: *Eg, patient.*  Participant #3 Type: *Eg, carer.* | | | Participant #4 Type: *Eg, additional (eg, Translator).*  Participant #5 Type: *Eg, additional (eg, social worker).* | | | |
| Construct 1: Eg, Patient safety.  Theory informed element #1 Eg, Medications.  Theory informed element #2  Theory informed element #3  Construct 2:  Theory informed element #1  Theory informed element #2  Theory informed element #3 | | | Other anticipated elements:  #1  #2  #3 | | | |
| *Construct 3 (Human-Computer Interaction):*  *Eg, notes created in relation to the IS Success model to measure clinician-IT HCIs.*  *System quality: Eg, the video conferencing of the system did not function.*  *Information quality: _________________________________________________________*  *System use: _________________________________________________________*  *User satisfaction: _________________________________________________________*  *Individual impact: _________________________________________________________*  *Organizational impact: ________________________________________________________* | | | | | | |
| Definitions and other key instructions:  *Eg, unfamiliar terminology or terms.* | | | | | | |
| Open-ended, descriptive fieldnotes recorded here:  *Eg, further information to provide context on what is being observed.* | | | | | | |

Figure 1 Semi-structured Observation Template.
